# 65 Differences in Pain Experience by Race, Ethnicity, and Socioeconomic Status Among People with Burn Injury

**DOI:** 10.1093/jbcr/irad045.039

**Published:** 2023-05-15

**Authors:** Maiya Pacleb, Barclay Stewart, Gretchen Carrougher, Karen Kowalske, Samuel Mandell, Jeffrey Schneider, Julie Silver, Kara McMullen, Caitlin Orton, Stephen Sibbett, Nathaniel Ashford

**Affiliations:** University of Washington, Seattle, Washington; University of Washington, Seattle, Washington; University of Washington, Seattle, Washington; UT Southwestern Medical Center, Dallas, Dallas, Texas; Parkland Burn Center / UT Southwestern, Dallas, Texas; Spaulding Rehabilitation Hospital/Harvard Medical School/Rehabilitation Outcomes Center at Spaulding, Boston, Massachusetts; Harvard Medical School, Boston, Massachusetts; University of Washington, Seattle, Washington; University of Washington, Seattle, Washington; University of Washington, Seattle, Washington; University of Washington, Seattle, Washington; University of Washington, Seattle, Washington; University of Washington, Seattle, Washington; University of Washington, Seattle, Washington; UT Southwestern Medical Center, Dallas, Dallas, Texas; Parkland Burn Center / UT Southwestern, Dallas, Texas; Spaulding Rehabilitation Hospital/Harvard Medical School/Rehabilitation Outcomes Center at Spaulding, Boston, Massachusetts; Harvard Medical School, Boston, Massachusetts; University of Washington, Seattle, Washington; University of Washington, Seattle, Washington; University of Washington, Seattle, Washington; University of Washington, Seattle, Washington; University of Washington, Seattle, Washington; University of Washington, Seattle, Washington; University of Washington, Seattle, Washington; UT Southwestern Medical Center, Dallas, Dallas, Texas; Parkland Burn Center / UT Southwestern, Dallas, Texas; Spaulding Rehabilitation Hospital/Harvard Medical School/Rehabilitation Outcomes Center at Spaulding, Boston, Massachusetts; Harvard Medical School, Boston, Massachusetts; University of Washington, Seattle, Washington; University of Washington, Seattle, Washington; University of Washington, Seattle, Washington; University of Washington, Seattle, Washington; University of Washington, Seattle, Washington; University of Washington, Seattle, Washington; University of Washington, Seattle, Washington; UT Southwestern Medical Center, Dallas, Dallas, Texas; Parkland Burn Center / UT Southwestern, Dallas, Texas; Spaulding Rehabilitation Hospital/Harvard Medical School/Rehabilitation Outcomes Center at Spaulding, Boston, Massachusetts; Harvard Medical School, Boston, Massachusetts; University of Washington, Seattle, Washington; University of Washington, Seattle, Washington; University of Washington, Seattle, Washington; University of Washington, Seattle, Washington; University of Washington, Seattle, Washington; University of Washington, Seattle, Washington; University of Washington, Seattle, Washington; UT Southwestern Medical Center, Dallas, Dallas, Texas; Parkland Burn Center / UT Southwestern, Dallas, Texas; Spaulding Rehabilitation Hospital/Harvard Medical School/Rehabilitation Outcomes Center at Spaulding, Boston, Massachusetts; Harvard Medical School, Boston, Massachusetts; University of Washington, Seattle, Washington; University of Washington, Seattle, Washington; University of Washington, Seattle, Washington; University of Washington, Seattle, Washington; University of Washington, Seattle, Washington; University of Washington, Seattle, Washington; University of Washington, Seattle, Washington; UT Southwestern Medical Center, Dallas, Dallas, Texas; Parkland Burn Center / UT Southwestern, Dallas, Texas; Spaulding Rehabilitation Hospital/Harvard Medical School/Rehabilitation Outcomes Center at Spaulding, Boston, Massachusetts; Harvard Medical School, Boston, Massachusetts; University of Washington, Seattle, Washington; University of Washington, Seattle, Washington; University of Washington, Seattle, Washington; University of Washington, Seattle, Washington; University of Washington, Seattle, Washington; University of Washington, Seattle, Washington; University of Washington, Seattle, Washington; UT Southwestern Medical Center, Dallas, Dallas, Texas; Parkland Burn Center / UT Southwestern, Dallas, Texas; Spaulding Rehabilitation Hospital/Harvard Medical School/Rehabilitation Outcomes Center at Spaulding, Boston, Massachusetts; Harvard Medical School, Boston, Massachusetts; University of Washington, Seattle, Washington; University of Washington, Seattle, Washington; University of Washington, Seattle, Washington; University of Washington, Seattle, Washington; University of Washington, Seattle, Washington; University of Washington, Seattle, Washington; University of Washington, Seattle, Washington; UT Southwestern Medical Center, Dallas, Dallas, Texas; Parkland Burn Center / UT Southwestern, Dallas, Texas; Spaulding Rehabilitation Hospital/Harvard Medical School/Rehabilitation Outcomes Center at Spaulding, Boston, Massachusetts; Harvard Medical School, Boston, Massachusetts; University of Washington, Seattle, Washington; University of Washington, Seattle, Washington; University of Washington, Seattle, Washington; University of Washington, Seattle, Washington; University of Washington, Seattle, Washington; University of Washington, Seattle, Washington; University of Washington, Seattle, Washington; UT Southwestern Medical Center, Dallas, Dallas, Texas; Parkland Burn Center / UT Southwestern, Dallas, Texas; Spaulding Rehabilitation Hospital/Harvard Medical School/Rehabilitation Outcomes Center at Spaulding, Boston, Massachusetts; Harvard Medical School, Boston, Massachusetts; University of Washington, Seattle, Washington; University of Washington, Seattle, Washington; University of Washington, Seattle, Washington; University of Washington, Seattle, Washington; University of Washington, Seattle, Washington; University of Washington, Seattle, Washington; University of Washington, Seattle, Washington; UT Southwestern Medical Center, Dallas, Dallas, Texas; Parkland Burn Center / UT Southwestern, Dallas, Texas; Spaulding Rehabilitation Hospital/Harvard Medical School/Rehabilitation Outcomes Center at Spaulding, Boston, Massachusetts; Harvard Medical School, Boston, Massachusetts; University of Washington, Seattle, Washington; University of Washington, Seattle, Washington; University of Washington, Seattle, Washington; University of Washington, Seattle, Washington; University of Washington, Seattle, Washington; University of Washington, Seattle, Washington; University of Washington, Seattle, Washington; UT Southwestern Medical Center, Dallas, Dallas, Texas; Parkland Burn Center / UT Southwestern, Dallas, Texas; Spaulding Rehabilitation Hospital/Harvard Medical School/Rehabilitation Outcomes Center at Spaulding, Boston, Massachusetts; Harvard Medical School, Boston, Massachusetts; University of Washington, Seattle, Washington; University of Washington, Seattle, Washington; University of Washington, Seattle, Washington; University of Washington, Seattle, Washington

## Abstract

**Introduction:**

Disparities in pain experience and treatment amongst people of different races and ethnicities have been described for several conditions. However, the relationship between race, ethnicity, socioeconomic status (SES) and pain reported by people with burn injury is not well understood. This study compares pain intensity and interference with daily activities among burn-injured adults of various sociodemographic backgrounds. We hypothesized that minority and low-income populations will report greater pain intensity and interference, necessitating additional strategies to address pain disparity.

**Methods:**

Adult multicenter national database participants with complete PROMIS^®^ pain intensity and pain interference measures at 6 and 12 months after injury were analyzed. Linear regression models examined associations between sex, race, ethnicity, education, income, burn size and pain interference and intensity scores. Regression model diagnostics were tested, and final models used robust standard errors to account for heteroskedasticity.

**Results:**

Data from 656 participants were analyzed, with a mean age of 47.1 ± 16.2. Racial representation was 84.0% White, 8.8% African American/Black, 2.5% Asian/Native Hawaiian/Pacific Islander, 1.9% American India laskan Native, and 2.8% other/more than one race; 80.4% were non-Hispanic and 19.6% Hispanic. Weighted regression models revealed that pain intensity at 6 mo (Ⓡ=1.25, p=0.029) and interference at 12 mo (β=6.71, p=0.013) among Black participants were markedly higher than White participants. Hispanic participants reported lower pain intensity (β=-0.86, p=0.036) and interference (β=-4.06, p=0.007) compared to non-Hispanic participants at 6 mo. Average pain intensity at 6 mo varied significantly by income (p=0.01), with the highest pain intensity (mean 3.8, SD 3.0) reported by those making < $25,000/year. Females reported greater pain intensity at 6 mo than males (β=0.63, p=0.029).

**Conclusions:**

Greater pain intensity and interference were reported by Black participants compared to White participants, while Hispanic participants reported lower pain outcomes. Lower income was also associated with worse pain outcomes. Targeted study of pre-injury pain experiences, pain management, psychosocial health, and financial toxicity are required to identify opportunities for intervention on the many dimensions and causal factors of unsatisfactory pain experiences.

**Applicability of Research to Practice:**

Systematic screening for pain intensity and interference after burn injury may find sociodemographic groups with high pain levels and a need for interdisciplinary pain and psychosocial health management. Comprehensive pain treatment following burn injury should account for social determinants of health and impact of bias to further improve quality care for all people.

